# Identification of a Somatic Mutation-Derived Long Non-Coding RNA Signatures of Genomic Instability in Renal Cell Carcinoma

**DOI:** 10.3389/fonc.2021.728181

**Published:** 2021-10-05

**Authors:** Xisheng Fang, Xia Liu, Lin Lu, Guolong Liu

**Affiliations:** ^1^ Department of Medical Oncology, Guangzhou First People’s Hospital, School of Medicine, South China University of Technology, Guangzhou, China; ^2^ Department of Medical Oncology, Guangzhou First People’s Hospital, Guangzhou Medical University, Guangzhou, China

**Keywords:** renal cell carcinoma, lncRNA, genomic instability, genome instability-related lncRNAs (GInLncRNAs), prognostic risk model

## Abstract

**Background:**

Renal cell carcinoma (RCC) is a malignant tumor with high morbidity and mortality. It is characterized by a large number of somatic mutations and genomic instability. Long non-coding RNAs (lncRNAs) are widely involved in the expression of genomic instability in renal cell carcinoma. But no studies have identified the genome instability-related lncRNAs (GInLncRNAs) and their clinical significances in RCC.

**Methods:**

Clinical data, gene expression data and mutation data of 943 RCC patients were downloaded from The Cancer Genome Atlas (TCGA) database. Based on the mutation data and lncRNA expression data, GInLncRNAs were screened out. Co-expression analysis, Gene Ontology (GO) enrichment analysis and Kyoto Encyclopedia of Genes and Genomes (KEGG) enrichment analysis were conducted to explore their potential functions and related signaling pathways. A prognosis model was further constructed based on genome instability-related lncRNAs signature (GInLncSig). And the efficiency of the model was verified by receiver operating characteristic (ROC) curve. The relationships between the model and clinical information, prognosis, mutation number and gene expression were analyzed using correlation prognostic analysis. Finally, the prognostic model was verified in clinical stratification according to TCGA dataset.

**Results:**

A total of 45 GInLncRNAs were screened out. Functional analysis showed that the functional genes of these GInLncRNAs were mainly enriched in chromosome and nucleoplasmic components, DNA binding in molecular function, transcription and complex anabolism in biological processes. Univariate and Multivariate Cox analyses further screened out 11 GInLncSig to construct a prognostic model (AL031123.1, AC114803.1, AC103563.7, AL031710.1, LINC00460, AC156455.1, AC015977.2, ‘PRDM16-dt’, AL139351.1, AL035661.1 and LINC01606), and the coefficient of each GInLncSig in the model was calculated. The area under the curve (AUC) value of the ROC curve was 0.770. Independent analysis of the model showed that the GInLncSig model was significantly correlated with the RCC patients’ overall survival. Furthermore, the GInLncSig model still had prognostic value in different subgroups of RCC patients.

**Conclusion:**

Our study preliminarily explored the relationship between genomic instability, lncRNA and clinical characteristics of RCC patients, and constructed a GInLncSig model consisted of 11 GInLncSig to predict the prognosis of patients with RCC. At the same time, our study provided theoretical support for the exploration of the formation and development of RCC.

## Introduction

As the most common urinary malignancy in the United States, there are about 73,820 new cases of RCC each year, and 14,770 deaths from the disease (1). At present, surgical resection is the main treatment for patients with RCC (2, 3). However, about 1/3 of patients have metastases at the time of diagnosis, and 1/5 patients have metastases or recurrences after radical treatment (4). RCC has a poor sensitivity to radiotherapy and chemotherapy. Targeted therapy and immunotherapy are the other treatment options (5–7). Currently, there is no biomarker in clinical practice that can well predict the prognosis of RCC patients.

In recent years, scientists have found that an important part of the non-coding genome is transcribed to produce non-coding RNAs, among which a subset longer than 200 nt at length, capped polyglandular transcripts transcribed by RNA polymerase II, which are known as long non-coding RNAs (lncRNA). It has been confirmed that lncRNA plays an important role in genomic function and gene expression, as well as in the etiology and treatment of malignant tumors (8). Some researchers have found that several lncRNAs are related to the prognosis of RCC patients. LncRNA B7H4 was expressed in the endothelium of tumor cells or tumor blood vessels but not in normal tissues. Therefore, low expression of B7H4 can be used as a predictor of survival in patients with RCC (9–11). TRAF3IP2-AS1 functioned as a tumor suppressor in RCC progression. Overexpression of TRAF3IP2-AS1 inhibited the proliferation, migration and invasion of UOK109 cells (12). Generally, single biomarkers have their limitations in terms of sensitivity and specificity. Therefore, some researchers have tried to construct prognostic models to predict the prognosis of RCC patients in a broader sense by screening a panel of biomarkers (13, 14).

Genomic instability plays a key role in the development of cancer. For example, structural changes in proto-oncogenes and tumor suppressor genes may lead to abnormalities in cell functions, including cell growth, cell cycle, cell senescence and apoptosis, cell invasion and metastasis (15–18). The genomic instabilities of cancer mainly include gene mutation, chromosome rearrangement and aneuploidy (19). Genomic instability has been identified as a key prognostic factor, and it is of great importance to explore its clinical significance.

## Materials and Methods

### Clinical Data Download and Data Consolidation

Gene transcriptome profiling, gene mutation data and clinical information of RCC patients were downloaded from the TCGA database as of March 2021. Perl software was used to extract all the mRNAs and lncRNAs and then annotated them with the HUGO Gene Nomenclature Committee (HGNC) database. This study complied with the publication guidelines of TCGA. And no additional ethical consent was required.

### Screening and Identification of lncRNAs Related to Genomic Instability

We extracted the lncRNA expression profile and the cumulative mutation frequency of each sample from the entire annotated transcriptome data. Wilcoxon rank sum test was used to screen GInLncRNAs differentially expressed between the top 25% of samples and the bottom 25% of samples with cumulative mutation frequency by using “limma” package in R software. Heat maps of differentially expressed GInLncRNAs in the two groups were constructed using the “pheatmap” package in the R software. Subsequently, the expression level of each sample was calculated according to the above GInLncRNAs and were divided into two groups. Median mutation frequency values of the two groups were calculated respectively. The two groups with higher and lower median values were set as Genomic Unstable type (GU-like) and Genomic Stable type (GS-like), respectively. Heatmaps of the two groups of differentially expressed lncRNAs were constructed using the “pheatmap” package in the R software. Next, the mutation frequency of all samples in the GU-like group and the GS-like group as well as the expression level of the genomic instability driver gene “UBQLN4” (20) were compared. Then the “ggpubr” package was used to construct a boxplot of the difference in mutation frequency and expression of “UBQLN4” gene between the two groups in R software.

### Gene Co-Expression Network

The expression levels of lncRNA and mRNA were retrieved using the “limma” package in R software. Pearson correlation analysis was performed to determine the target mRNA of GInLncRNA. The top ten genes with the highest correlation were selected as the target genes of this GInLncRNA according to Pearson correlation coefficient, and the co-expression network was visualized by Cytoscape software.

### Functional Enrichment Analysis

In order to predict the potential function and pathway of the GInLncRNAs, we performed functional enrichment analysis on related mRNAs co-expressed with GInLncRNAs to determine significantly enriched GO terms and KEGG pathways. The packages of “clusterProfiler”, “org.Hs.eg.db”, “enrichplot” and “ggplot2” in R software were used with P value and adjust P value < 0.05.

### Construction of a Prognostic Risk Model

After matching the expression of lncRNA transcriptional profile, somatic mutation data and clinicopathological features of the RCC patients, we randomly divided 857 samples into two groups in a ratio of 1:1 and named Train set and Test set, using the “caret” package in R software. The 429 samples of the Train set were designed to identify GInLncSig and to construct prognostic model. Besides, the 428 samples of the Test set were used to investigate the performance of the prognostic model. Univariate Cox analysis was conducted to find out the GInLncSig in the Train set with the “Survival” package in R software. Subsequently these GInLncSig were used to construct a prognostic model.

### Assessment of the Performance of the Prognostic Risk Model

Each patient’s risk value in the Train set was calculated by the coef of GInLncSig in the prognostic model. According to the median risk value of the Train set, patients in the Test set and entire TCGA cohort were divided into high-risk group and low-risk group. The packages “survival” and “survminer” were used to perform log-rank test (P <0.05) for patients in the high-risk and low-risk groups in R software. Furthermore, the Kaplan-Meier method was used to draw the survival curves of the Test set and entire TCGA cohort. The Receiver Operating Characteristic (ROC) curve with Areas Under Curve (AUC) values of the prognostic model was assessed by the “survival ROC” package in R software.

### Independent Analysis of the Model Constructed by GInLncSig in Clinical Stratification

In order to test whether the prognostic model constructed by GInLncSig can be used as an independent prognostic factor, we extracted the clinical information of RCC patients in TCGA database and deleted the cases with clinical information gaps. Univariate Cox regression analysis was performed to explore the prognostic values of age, gender, tumor grade, tumor clinical stage and prognostic model using the “survival” package of R software. And Multivariate prognostic analysis was performed for factors with P < 0.05 in the extraction results. Next, we divided the RCC patients of the TCGA database into different subgroups according to clinical parameters, including age (≦ 65 years and > 65), gender (female and male), tumor grade (G1-2 and G3-4), and tumor stage (I-II and III-IV). Patients in each clinical subgroup were further divided into high-risk and low-risk groups based on the median risk value. The Kaplan- Meier analysis and log-rank test were used to compare the differences in survival between high-risk and low-risk groups in each clinical subgroup.

### Statistical Analysis

Gene expression, mutation data and clinical information from the TCGA dataset were retrieved in R software (R3.6.2) or Perl software (Strawberry Perl(64-bit)). All the statistical analyses were evaluated by R software. AP-value < 0.05 was statistically significant.

## Results

### Screening of Genomic Instability-Related lncRNAs of RCC Patients in TCGA Database

**Figure 1:**
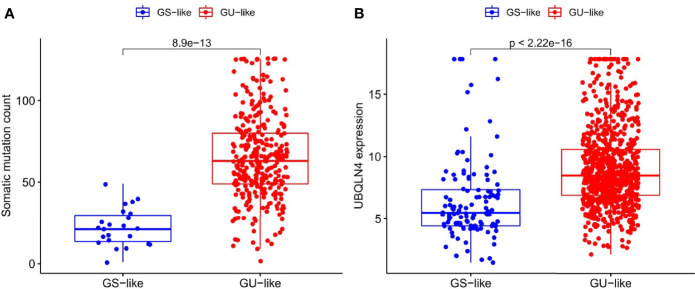
Differences in somatic mutation counts and UBQLN4 between GU-like group and GS-like group. **(A)** Differences in somatic mutation counts between GU-like group and GS-like group. The mutation counts of the GS-like group were significantly lower than that of the GU-like group (P < 0.001, Mann–Whitney U test). **(B)** Differences in expression of UBQLN4 between GU-like group and GS-like group. The expression of UBQLN4 in GU-like group were significantly higher than that of the GS-like group (P < 0.001, Mann–Whitney U test).

The somatic mutation information of 336 patients with RCC, lncRNA and mRNA transcriptional profiles of 895 patients with RCC, and clinicopathological features of 943 patients with RCC were downloaded from the TCGA database. Each sample was ranked according to the number of mutations. There were 45 GInLncRNAs differentially expressed between the GU-like group and GS-like group. All the differentially expressed GInLncRNAs were demonstrated by heat maps (**Supplementary Figure 1**). Subsequently, boxplot showed that there were significant differences in the somatic mutation count (**Figure 1A**) and the expression of genomic instability driver gene “UBQLN4” (**Figure 1B**) between the two groups.

### Analysis of Potential Functions and Pathways of the GInLncRNAs

**Figure 2:**
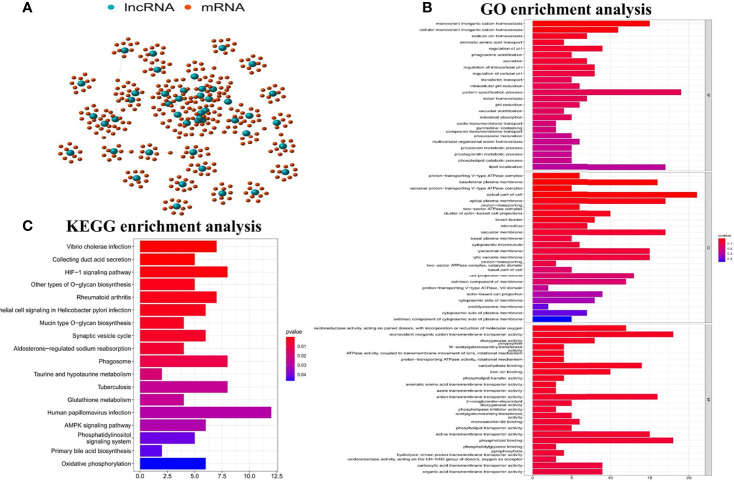
Functional analyses of the GInLncRNAs. **(A)** The relationship networks between GInLncRNAs and their top 10 co-expressed protein-coding genes were constructed according to Pearson correlation coefficient. The GInLncRNAs and protein-coding mRNAs were represented by blue and orange circles, respectively. **(B)** Barplot of GO enrichment analysis of protein genes co-expressed with the GInLncRNAs(P<0.05). **(C)** Barplot of KEGG enrichment analysis of protein genes co-expressed with the GInLncRNAs (P<0.05).

In order to determine whether the functions and pathways of these 45 differential GInLncRNAs were related to genomic instability, we explored the potential functions of GInLncRNAs by co-expression analysis, GO function enrichment analysis and KEGG pathway analysis. **Figure 2A** showed the co-expression network of IncRNA-mRNA which reflecting the relationship between them. The names of the top 10 mRNAs co-expressed with each GInLncRNA were labeled according to Pearson correlation coefficient analysis. GO function enrichment analysis of GInLncRNA-related genes showed that their functions were mainly enriched in chromosome and nucleoplasmic components (CC), DNA binding in molecular function (MF), and transcription and complex anabolism in biological processes (BP) (P<0.05) (**Figure 2B**). By analyzing the KEGG pathways of LncRNA-associated protein-related genes, 18 pathways were found to be significantly enriched. And most of the enriched pathways were related to genomic instability, including HIF-1 signaling pathway, AMPK signaling pathway and Oxidative phosphorylation (**Figure 2C**).

### Construction of a Prognostic Model

A total of 857 RCC patients in the TCGA database had complete lncRNA expression profiles, somatic mutation data and clinicopathological characteristics. **Table 1** showed that there were no significant differences in clinicopathological characteristics between Train set (429 cases) and Test set (428 cases) (including age, gender, tumor grade, clinical stage, T stage, N stage, and M stage).

**Table 1:** Clinicopathological information of the patients with RCC in TCGA cohort.

Covariates	Type	Total (n = 857)	Test (n = 428)	Train (n = 429)	Pvalue
Age	<=65	560 (65.34%)	283 (66.12%)	277 (64.57%)	0.5818
Age	>65	292 (34.07%)	141 (32.94%)	151 (35.2%)	
Age	unknown	5 (0.58%)	4 (0.93%)	1 (0.23%)	
Gender	FEMALE	274 (31.97%)	139 (32.48%)	135 (31.47%)	0.8079
Gender	MALE	583 (68.03%)	289 (67.52%)	294 (68.53%)	
Grade	G1-2	231 (26.95%)	109 (25.47%)	122 (28.44%)	0.4319
Grade	G3-4	274 (31.97%)	140 (32.71%)	134 (31.24%)	
Grade	unknown	352 (41.07%)	179 (41.82%)	173 (40.33%)	
Stage	Stage I-II	543 (63.36%)	271 (63.32%)	272 (63.4%)	1
Stage	Stage III-IV	281 (32.79%)	140 (32.71%)	141 (32.87%)	
Stage	unknown	33 (3.85%)	17 (3.97%)	16 (3.73%)	
T	T1-2	592 (69.08%)	294 (68.69%)	298 (69.46%)	0.7837
T	T3-4	261 (30.46%)	133 (31.07%)	128 (29.84%)	
T	unknown	4 (0.47%)	1 (0.23%)	3 (0.7%)	
M	M0	532 (62.08%)	267 (62.38%)	265 (61.77%)	0.7314
M	M1	89 (10.39%)	47 (10.98%)	42 (9.79%)	
M	unknown	236 (27.54%)	114 (26.64%)	122 (28.44%)	
N	N0	312 (36.41%)	153 (35.75%)	159 (37.06%)	0.2965
N	N1-2	48 (5.6%)	28 (6.54%)	20 (4.66%)	
N	unknown	497 (57.99%)	247 (57.71%)	250 (58.28%)	

Chi-squared test, P < 0.05 means significantly different.

**Figure 3:**
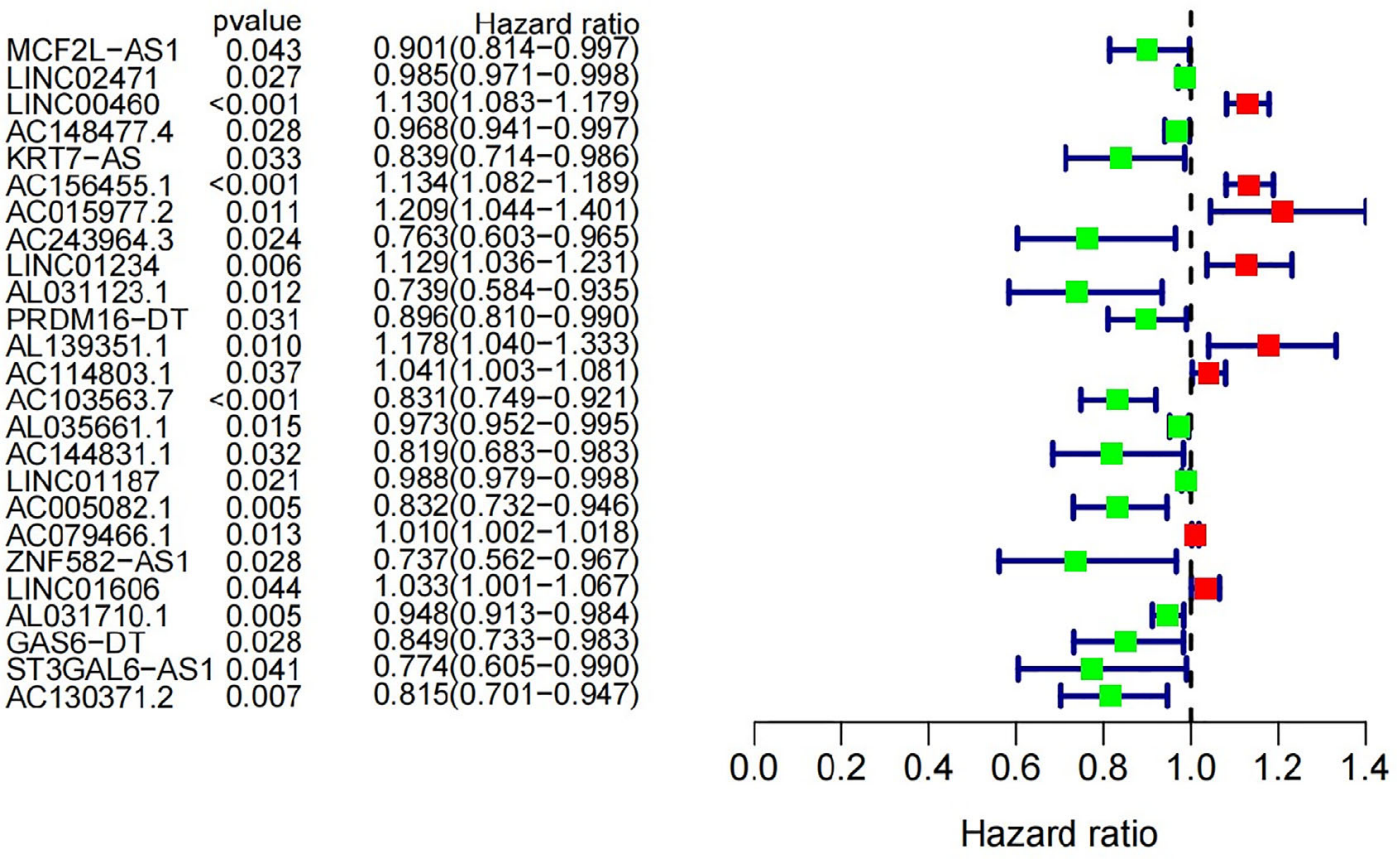
Univariate Cox regression analysis was used in the Train set to construct forest plot of 25 GInLncRNAs associated with patients’ overall survival. Eight GInLncRNAs were protecting factors for patients’ survival (LINC00460, AC156455.1, AC015977.2, LINC01234, AL139351.1, AC114803.1, AC079466.1 and LINC01606), while the other seventeen GInLncRNAs were the risk factors for patients’ survival.

Univariate Cox proportional hazard regression analysis was used for screening. And 25 out of 45 GInLncRNAs in the Train set were significantly associated with overall survival (P<0.05, **Figure 3** and **Supplementary Table 1**). Subsequently, a survival prediction model with 11 GInLncRNAs was constructed by Multivariate Cox proportional hazard regression analysis (**Supplementary Table 2**). Among them, the coefficient of four GInLncRNAs (AL031123.1, AC114803.1, AC103563.7 and AL031710.1) were negative, indicating that they might act as protective factors. Up-regulated expression of them were associated with better survival. Otherwise, the coefficient of seven GInLncRNAs (LINC00460, AC156455.1, AC015977.2, ‘PRDM16-dt’, AL139351.1, AL035661.1, LINC01606) were positive, indicating that they were risk factors and up-regulated expression of them were associated with poor prognosis.

### Assessment of the Prognostic Model

**Figure 4:**
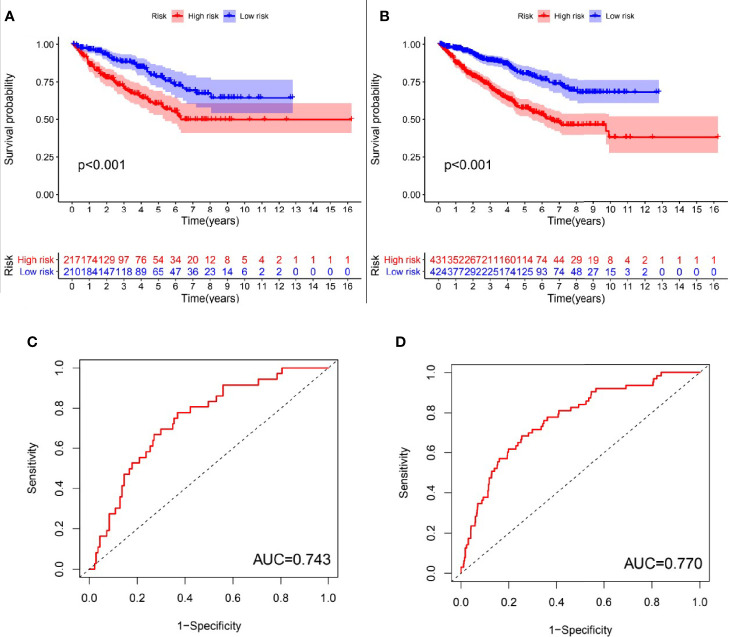
Evaluation of the predictive performance of GInLncSig’s for overall survival in patients with RCC. Kaplan-Meier survival curve **(A, B)** showed that there was significant difference in the survival rate between the high-risk group and low-risk group in the Test set and TCGA set, respectively (log-rank test, p < 0.05). ROC curves for 1-year survival prediction of the GInLncSig in the Test set **(C)** and the TCGA set **(D)**.

In order to evaluate the predictive effectiveness of the prognostic model, patients in the Test set and entire TCGA cohort were divided into high-risk group and low-risk group according to the median risk value of patients in the Train set. It was obvious that the overall survival of patients in the low-risk group was significantly better than the high-risk group(P<0.001, log-rank test; **Figures 4A, B**). The AUC of the ROC curves in the Test set and entire TCGA cohort were 0.743 and 0.770, respectively, which suggesting that the risk model had a good predictive effectiveness (**Figures 4C, D**).

### Independent Analysis of the Prognostic Model in Clinical Stratification

To explore whether the prognostic model constructed by GInLncSig can be used as an independent prognostic factor, Univariate and Multivariate Cox regression analyses were performed to analyze the prognostic values of age, gender, tumor grade, tumor clinical stage and the prognostic model. As shown in **Table 2**, clinical information, including age, gender, tumor grade, tumor clinical stage as well as the prognostic model were significantly correlated with the patients’ overall survival in univariate Cox analysis. And Multivariate Cox analysis showed that age, tumor grade, tumor clinical stage and the prognostic model still retained prognostic significance.

**Table 2:** Univariate and Multivariate Cox regression analysis of the prognostic model and overall survival in TCGA set.

Variables	Univariable model				Multivariable model			
	HR	HR.95L	HR.95H	pvalue	HR	HR.95L	HR.95H	pvalue
Age	1.029	1.015	1.042	<0.001	1.031	1.016	1.046	<0.001
Gender	0.964	0.703	1.323	0.821				
Grade	2.268	1.845	2.787	<0.001	1.466	1.163	1.848	0.001
Stage	1.896	1.658	2.168	<0.001	1.669	1.433	1.945	<0.001
GInLncSig model	1.026	1.016	1.036	<0.001	1.012	1.001	1.022	0.037

**Figure 5:**
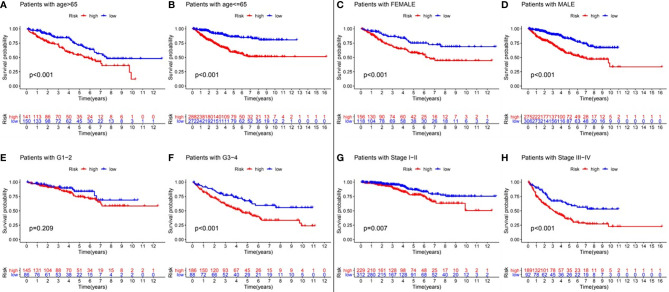
Clinical stratification analysis of the differences in overall survival between the high- and low-risk RCC patients by age, gender, tumor grade and tumor clinical stage. Kaplan-Meier survival curves of patients in high- and low-risk groups within six clinically stratified subgroups, including patients with age>65 years **(A)**, age<=65 years **(B)**, the gender of female **(C)**, the gender of male **(D)**, the tumor grade of G1-2 **(E)**, grade of G3-4 **(F)**, the tumor stage of I-II **(G)**, and the tumor stage of III-IV **(H)**, respectively. Log-rank test (p<0.05) showed that patients with a low-risk score had significantly better survival outcomes than those with a high-risk score in all the subgroups except for the tumor grade G1-2 subgroup.

In order to verify whether the prognostic model still had prognostic value in different subgroups of RCC patients, we divided the RCC patients into different subgroups according to various clinical parameters, including age (≦ 65 years and > 65 years), gender (female and male), tumor grade (G1-2 and G3-4), and tumor stage (I-II and III-IV). Kaplan-Meier analysis showed that patients with a low-risk value had significantly better survival outcomes than those with a high-risk value in all the subgroups except for the G1-2 subgroup (**Figure 5**). These results suggested that the model constructed by GInLncSig can be used as an independent prognostic factor for different subgroups of RCC patients.

## Discussion

Genomic instability was a characteristic feature of most human cancers (21). Generally, genomic instability included single-base, double-base, or cluster-base substitutions, copy number changes, small insertions and deletions, and genome rearrangements. The accumulation of genetic changes could turn normal cells into malignant cells (22–24). In addition, persistent genomic instability allowed tumor cells to adapt to their microenvironment under selective pressure and thus become resistant to antitumor therapies (25, 26). Processes of genomic instability drove tumors genetics. Studies had found that proto-oncogenes and tumor suppressor genes in nearly 20 common malignant tumors, in which Kidney chromophobe (KICH) was the fewest with 2 genes and Uterine Corpus Endometrial Carcinoma (UCEC) were the most with 55 genes (22). Furthermore, genomic instability was widely involved in the diagnosis and prognosis of various malignant tumors, especially esophageal, bladder, breast, and lung cancers (15, 17, 27, 28). Long non-coding RNAs (lncRNAs) were major components of the mammalian transcriptome and played central roles in a variety of cellular mechanisms. At present, there was evidence indicating that lncRNA was closely related to genomic instability (29). It could promote the occurrence, development and metastasis of RCC (30–32). However, there were still few studies concerning the relationship between lncRNA associated with genomic instability in RCC, which was the main purpose of this research.

In our study, 45 lncRNAs were identified by comparing the expression levels of lncRNAs between genetic-unstable and genetic-stable RCC tumors from the selected patients based on TCGA database. Co-expression analysis, GO enrichment analysis and KEGG pathway analysis showed that the genes co-expressed with these 45 lncRNAs mainly enriched in pathways including HIF-1 signaling pathway, AMPK signaling pathway and Oxidative phosphorylation signaling pathway, which had also been proven to be related to genomic instability of tumors (33–35). These results suggested that the differentially expressed GInLncRNAs may have direct or indirect causal relationships with genomic instability through the above mentioned pathways. Next, Univariate Cox regression analysis was used to screen 25 GInLncRNAs that were significantly related to the survival of RCC patients from the 45 differentials expressed GInLncRNAs in the Train set. Moreover, Multivariate Cox regression analysis was used to screen 11 GInLncRNAs from the 25 prognostic GInLncRNAs to build a prognostic model. The correlation coefficient calculation formula of the prognostic model for each GInLncSig was obtained, and the risk value of each patient in the Test set was calculated according to the prognostic model. The results showed that patients with high risk value tended to have poor overall survival. The AUC of the model was > 0.7 in both the Train set and the Test set, which indicating that the model had good prediction performance. According to Multivariate Cox regression analysis, we found that the prognostic model had independent prognostic value as well as age, tumor grade and tumor clinical stage. Furthermore, the prognostic model had prognostic value in patients with different ages (≦ 65 years and > 65 years), gender (female and male), tumor grade (G3-4) and clinical stage (I-II and III-IV). These results enabled the GInLncSig model to have a wider range of application, and which could well predict the prognosis of RCC patients in different stratification.

Scholars had explored some prognostic models in RCC. May et al. predicted the disease-free and tumor-related survival rate of patients with RCC undergoing reoperation by using three pathological factors of tumor microvascular invasion, size and grade (36). Later studies gradually moved to the detection of molecules in the blood, including tumor markers (37), protein expression (38), and RNA-binding proteins (39). Some research also explored corresponding prognostic models according to metastatic (40–42) and non-metastatic (43, 44) RCC. With the development of next-generation sequencing (NGS) technologies, the detection of DNA, microRNA and lncRNA in the blood or tissues of patients had become more and more convenient and accessible. There were also studies that systematically evaluated the expression of DNA methylation markers (45), microRNA (46), and lncRNA (47, 48) to predict the prognosis of patients with RCC. However, these models were not relate to genomic instability, and some models could only predict the prognosis of specific patients, such as metastatic or non-metastatic RCC. In contrast, our prognostic model constructed by using genomic instability could not only effectively predict the prognosis of all patients with RCC, but also had predictive value for patients with different stratification.

Some of the GInLncSig in our model had also been verified to be abnormally expressed in a variety of malignant tumors and had prognostic values. LINC00460 was located on chromosome 13q33.2 and was transcribed as a 935nt transcript (49). Currently, abnormal expression of LINC00460 had been found in many cancers, including nasopharyngeal carcinoma (50), esophageal squamous cell carcinoma (51), colorectal carcinoma (52–54), lung adenocarcinoma (49), non-small cell lung cancer (55–57), etc. Upregulated expression of LINC00460 predicted poorer differentiation, advanced stage, and worse overall survival (58). In addition, mechanism studies had shown that overexpression of LINC00460 may promote invasion and metastasis of NSCLC cells through epithelial-mesenchymal transformation pathway (57). Data had shown that LINC01606 was significantly overexpressed in gastric cancer cells compared with normal cells, and it promoted the migration and invasion of gastric cancer cells by acting as a ceRNA of miR-423-5p in Wnt/β-catenin-dependent pathway (59). This suggested that LINC01606 may act as an oncogene in gastric cancer. Zimta et al. found that the expression of PRDM16-DT was down-regulated in acute myeloid leukemia, and patients with lower expression of PRDM16-DT had better prognosis than those with higher expression (60).

This is a preliminary study that explores the relationship between genomic instability, lncRNA and clinical characteristics of RCC patients. We constructed a GInLncSig model, which could provide prognostic information in different clinical stratification. However, there were still several limitations of our study. First of all, the data sources of this study were only limited to TCGA database, and had not been further verified in multiple databases or clinical data of our hospital. Secondly, it was necessary to further explore the respective functions of each lncRNA in the prognostic model. Finally, given the NGS is becoming more available, consequently changes in gene expression are expected to be monitored during the treatment in the future.

## Data Availability Statement

The original contributions presented in the study are included in the article/**Supplementary Material**. Further inquiries can be directed to the corresponding authors.

## Author Contributions

GL and LL designed the study. XF performed the data analysis, graphing, and writing. XL was responsible for the critical reading of the manuscript. All authors contributed to the article and approved the submitted version.

## Funding

This research was funded by the Guangdong Medical Science and Research Foundation (No. B2021023), Guangzhou Planned Project of Science and Technology (No. 202102080609, No. 201904010427), the Natural Science Foundation of Guangdong Province, China (No. 2021A1515011113).

## Conflict of Interest

The authors declare that the research was conducted in the absence of any commercial or financial relationships that could be construed as a potential conflict of interest.

## Publisher’s Note

All claims expressed in this article are solely those of the authors and do not necessarily represent those of their affiliated organizations, or those of the publisher, the editors and the reviewers. Any product that may be evaluated in this article, or claim that may be made by its manufacturer, is not guaranteed or endorsed by the publisher.

## Abbreviations

RCC, Renal cell carcinoma; lncRNAs, Long non-coding RNAs; GInLncRNAs, genome instability-related lncRNAs; TCGA, The Cancer Genome Atlas; GO, Gene Ontology; KEGG, Kyoto Encyclopedia of Genes and Genomes; GInLncSig, genome instability-related lncRNA signature; ROC, receiver operating characteristic; AUC, area under the curve; HGNC, HUGO Gene Nomenclature Committee; GU-like, Genomic Unstable type; GS-like, Genomic Stable type; CC, chromosome and nucleoplasmic components; MF, molecular function; BP, biological processes; KICH, Kidney chromophobe; UCEC, Uterine Corpus Endometrial Carcinoma; NGS, next-generation sequencing.

## References

[1] Siegel RL Miller KD Jemal A. Cancer Statistics, 2019. CA Cancer J Clin (2019) 69:7–34. doi: 10.3322/caac.21551 30620402

[2] Qi N Zhao F Liu X Wei W Wang J. Safety of Prolonged Wait Time for Nephrectomy for Clinically Localized Renal Cell Carcinoma. Front Oncol (2021) 11:617383. doi: 10.3389/fonc.2021.617383 33859936 PMC8042291

[3] Saito Z Hata K Nishioka S Tamura K Tamura N Yoshida M. Localized Pleural Metastasis Without Other Organ Metastases After Nephrectomy for Renal Cell Carcinoma. Respir Med Case Rep (2021) 33:101388. doi: 10.1016/j.rmcr.2021.101388 33854938 PMC8025045

[4] Abu-Ghanem Y Powles T Capitanio U Beisland C Jarvinen P Stewart GD. Should Patients With Low Risk Renal Cell Carcinoma be Followed Differently After Nephron-Sparing Surgery Versus Radical Nephrectomy? BJU Int (2021) 128(3):386–94. doi: 10.1111/bju.15415 33794055

[5] Liu Y Zhang Z Han H Guo S Liu Z Liu M. Survival After Combining Stereotactic Body Radiation Therapy and Tyrosine Kinase Inhibitors in Patients With Metastatic Renal Cell Carcinoma. Front Oncol (2021) 11:607595. doi: 10.3389/fonc.2021.607595 33692951 PMC7937906

[6] Paciotti M Schmidt A Ravi P McKay R Trinh Q Choueiri T. Temporal Trends and Predictors in the Use of Stereotactic Body Radiotherapy for Treatment of Metastatic Renal Cell Carcinoma in the U.S. Oncol (2021) 26:e905–906. doi: 10.1002/onco.13736 PMC8100555 33650184

[7] Acharya N Singh K. Differential Sensitivity of Renal Carcinoma Cells to Doxorubicin and Epigenetic Therapeutics Depends on the Genetic Background. Mol Cell Biochem (2021) 476:2365–79. doi: 10.1007/s11010-021-04076-7 33591455

[8] Flippot R Beinse G Boilève A Vibert J Malouf G. Long non-Coding RNAs in Genitourinary Malignancies: A Whole New World. Nat Rev Urol (2019) 16:484–504. doi: 10.1038/s41585-019-0195-1 31110275

[9] Harshman LC Choueiri TK Drake C Stephen Hodi F Jr. Subverting the B7-H1/PD-1 Pathway in Advanced Melanoma and Kidney Cancer. Cancer J (2014) 20:272–80. doi: 10.1097/PPO.0000000000000055 PMC4894310 25098288

[10] Thompson RH Gillett MD Cheville JC Lohse CM Dong H Webster WS. Costimulatory B7-H1 in Renal Cell Carcinoma Patients: Indicator of Tumor Aggressiveness and Potential Therapeutic Target. Proc Natl Acad Sci USA (2004) 101:17174–9. doi: 10.1073/pnas.0406351101 PMC534606 15569934

[11] Thompson RH Kwon ED. Significance of B7-H1 Overexpression in Kidney Cancer. Clin Genitourin Cancer (2006) 5:206–11. doi: 10.3816/CGC.2006.n.038 17239274

[12] Yang L Chen Y Liu N Shi Q Han X Gan W. Low Expression of TRAF3IP2-AS1 Promotes Progression of NONO-TFE3 Translocation Renal Cell Carcinoma by Stimulating N(6)-Methyladenosine of PARP1 mRNA and Downregulating PTEN. J Hematol Oncol (2021) 14:46. doi: 10.1186/s13045-021-01059-5 33741027 PMC7980631

[13] Gao S Gao L Wang S Shi X Yue C Wei S. ATF3 Suppresses Growth and Metastasis of Clear Cell Renal Cell Carcinoma by Deactivating EGFR/AKT/GSK3beta/beta-Catenin Signaling Pathway. Front Cell Dev Biol (2021) 9:618987. doi: 10.3389/fcell.2021.618987 33816467 PMC8017234

[14] Chu G Xu T Zhu G Liu S Niu H Zhang M. Identification of a Novel Protein-Based Signature to Improve Prognosis Prediction in Renal Clear Cell Carcinoma. Front Mol Biosci (2021) 8:623120. doi: 10.3389/fmolb.2021.623120 33842538 PMC8027127

[15] Aguilar-Mahecha A Lafleur J Brousse S Savichtcheva O Holden K Faulkner N. Early, On-Treatment Levels and Dynamic Changes of Genomic Instability in Circulating Tumor DNA Predict Response to Treatment and Outcome in Metastatic Breast Cancer Patients. Cancers (2021) 13(6):1331. doi: 10.3390/cancers13061331 33809567 PMC7999382

[16] Dionellis V Norkin M Karamichali A Rossetti G Huelsken J Ordonez-Moran P. Genomic Instability Profiles at the Single Cell Level in Mouse Colorectal Cancers of Defined Genotypes. Cancers (2021) 13(6):1267. doi: 10.3390/cancers13061267 33809306 PMC7999300

[17] Geng W Lv Z Fan J Xu J Mao K Yin Z. Identification of the Prognostic Significance of Somatic Mutation-Derived LncRNA Signatures of Genomic Instability in Lung Adenocarcinoma. Front Cell Dev Biol (2021) 9:657667. doi: 10.3389/fcell.2021.657667 33855028 PMC8039462

[18] Motazedian A Dawson M. MSL Pushes Genomic Instability Over the Edge. Nat Cell Biol (2021) 23:295–6. doi: 10.1038/s41556-021-00666-1 33837286

[19] Klimaszewska-Wiśniewska A Buchholz K Neska-Długosz I Durślewicz J Grzanka D Zabrzyński J. Expression of Genomic Instability-Related Molecules: Cyclin F, RRM2 and SPDL1 and Their Prognostic Significance in Pancreatic Adenocarcinoma. Cancers (2021) 13(4):859. doi: 10.3390/cancers13040859 33670609 PMC7922901

[20] Jachimowicz R Beleggia F Isensee J Velpula B Goergens J Bustos M. UBQLN4 Represses Homologous Recombination and Is Overexpressed in Aggressive Tumors. Cell (2019) 176:505–19.e22. doi: 10.1016/j.cell.2018.11.024 30612738

[21] Yao Y Dai W. Genomic Instability and Cancer. J Carcinog Mutagen (2014) 5. doi: 10.4172/2157-2518.1000165 PMC4274643 25541596

[22] Bailey MH Tokheim C Porta-Pardo E Sengupta S Bertrand D Weerasinghe A. Comprehensive Characterization of Cancer Driver Genes and Mutations. Cell (2018) 174:1034–5. doi: 10.1016/j.cell.2018.07.034 PMC8045146 30096302

[23] Martinez-Jimenez F Muinos F Sentis I Deu-Pons J Reyes-Salazar I Arnedo-Pac C. A Compendium of Mutational Cancer Driver Genes. Nat Rev Cancer (2020) 20:555–72. doi: 10.1038/s41568-020-0290-x 32778778

[24] Alexandrov LB Kim J Haradhvala NJ Huang MN Tian Ng AW Wu Y. The Repertoire of Mutational Signatures in Human Cancer. Nature (2020) 578:94–101. doi: 10.1038/s41586-020-1943-3 32025018 PMC7054213

[25] Raghuram GV Chaudhary S Johari S Mittra I. Illegitimate and Repeated Genomic Integration of Cell-Free Chromatin in the Aetiology of Somatic Mosaicism, Ageing, Chronic Diseases and Cancer. Genes (Basel) (2019) 10(6):407. doi: 10.3390/genes10060407 PMC6628102 31142004

[26] Aguilera A Garcia-Muse T. Causes of Genome Instability. Annu Rev Genet (2013) 47:1–32. doi: 10.1146/annurev-genet-111212-133232 23909437

[27] Sihag S Nussenzweig S Walch H Hsu M Tan K Sanchez-Vega F. Next-Generation Sequencing of 487 Esophageal Adenocarcinomas Reveals Independently Prognostic Genomic Driver Alterations and Pathways. Clin Cancer Res An Off J Am Assoc Cancer Res (2021) 27(12):3491–8. doi: 10.1158/1078-0432.CCR-20-4707 PMC8228505 33795256

[28] Wu H Zhang Z Zhang Z Xiao X Gao S Lu C. Prediction of Bladder Cancer Outcome by Identifying and Validating a Mutation-Derived Genomic Instability-Associated Long Noncoding RNA (lncRNA) Signature. Bioengineered (2021) 12:1725–38. doi: 10.1080/21655979.2021.1924555 PMC8806732 33955803

[29] Lee S Kopp F Chang TC Sataluri A Chen B Sivakumar S. Noncoding RNA NORAD Regulates Genomic Stability by Sequestering PUMILIO Proteins. Cell (2016) 164:69–80. doi: 10.1016/j.cell.2015.12.017 26724866 PMC4715682

[30] Guo YP Wang ZF Li N Lei QQ Cheng Q Shi LG. Suppression of lncRNA HOTAIR Alleviates RCC Angiogenesis Through Regulating miR-126/EGFL7 Axis. Am J Physiol Cell Physiol (2021) 320(5):C880–91. doi: 10.1152/ajpcell.00459.2019 33502949

[31] Yang F Wu Q Zhang Y Xiong H Li X Li B. LncRNA LOC653786 Promotes Growth of RCC Cells via Upregulating FOXM1. Oncotarget (2018) 9:12101–11. doi: 10.18632/oncotarget.24027 PMC5844731 29552295

[32] Zhai W Sun Y Guo C Hu G Wang M Zheng J. LncRNA-SARCC Suppresses Renal Cell Carcinoma (RCC) Progression via Altering the Androgen Receptor(AR)/miRNA-143-3p Signals. Cell Death Differ (2017) 24:1502–17. doi: 10.1038/cdd.2017.74 PMC5563985 28644440

[33] Putra AC Tanimoto K Arifin M Hiyama K. Hypoxia-Inducible Factor-1alpha Polymorphisms are Associated With Genetic Aberrations in Lung Cancer. Respirology (2011) 16:796–802. doi: 10.1111/j.1440-1843.2011.01972.x 21435097

[34] Pascale RM Feo CF Calvisi DF Feo F. Deregulation of Methionine Metabolism as Determinant of Progression and Prognosis of Hepatocellular Carcinoma. Transl Gastroenterol Hepatol (2018) 3:36. doi: 10.21037/tgh.2018.06.04 30050996 PMC6044036

[35] Chan SSL. Inherited Mitochondrial Genomic Instability and Chemical Exposures. Toxicology (2017) 391:75–83. doi: 10.1016/j.tox.2017.07.014 28756246 PMC5681375

[36] May M Brookman-Amissah S Kendel F Knoll N Roigas J Hoschke B. Validation of a Postoperative Prognostic Model Consisting of Tumor Microvascular Invasion, Size, and Grade to Predict Disease-Free and Cancer-Specific Survival of Patients With Surgically Resected Renal Cell Carcinoma. Int J Urol (2009) 16:616–21. doi: 10.1111/j.1442-2042.2009.02319.x 19456988

[37] Kim HL Seligson D Liu X Janzen N Bui MH Yu H. Using Tumor Markers to Predict the Survival of Patients With Metastatic Renal Cell Carcinoma. J Urol (2005) 173:1496–501. doi: 10.1097/01.ju.0000154351.37249.f0 15821467

[38] Kim HL Seligson D Liu X Janzen N Bui MH Yu H. Using Protein Expressions to Predict Survival in Clear Cell Renal Carcinoma. Clin Cancer Res (2004) 10:5464–71. doi: 10.1158/1078-0432.CCR-04-0488 15328185

[39] Qin X Liu Z Yan K Fang Z Fan Y. Integral Analysis of the RNA Binding Protein-Associated Prognostic Model for Renal Cell Carcinoma. Int J Med Sci (2021) 18:953–63. doi: 10.7150/ijms.50704 PMC7807188 33456353

[40] Bearrick EN Packiam V Bhindi B Lohse CM Cheville JC Mason RJ. Creation of a Primary Tumor Tissue Expression Biomarker-Augmented Prognostic Model for Patients With Metastatic Renal Cell Carcinoma. Urol Oncol (2021) 39:135.e1–8. doi: 10.1016/j.urolonc.2020.08.028 33309297

[41] Kim HL Halabi S Li P Mayhew G Simko J Nixon AB. A Molecular Model for Predicting Overall Survival in Patients With Metastatic Clear Cell Renal Carcinoma: Results From CALGB 90206 (Alliance). EBioMedicine (2015) 2:1814–20. doi: 10.1016/j.ebiom.2015.09.012 PMC4740313 26870806

[42] Ohlmann CH. Prognostic Factors for Survival of Patients With Metastatic Renal Cell Carcinoma. Urol A (2009) 48:625–7. doi: 10.1007/s00120-009-1960-1 19300979

[43] Cindolo L de la Taille A Messina G Romis L Abbou CC Altieri V. A Preoperative Clinical Prognostic Model for non-Metastatic Renal Cell Carcinoma. BJU Int (2003) 92:901–5. doi: 10.1111/j.1464-410X.2003.04505.x 14632843

[44] Yaycioglu O Roberts WW Chan T Epstein JI Marshall FF Kavoussi LR. Prognostic Assessment of Nonmetastatic Renal Cell Carcinoma: A Clinically Based Model. Urology (2001) 58:141–5. doi: 10.1016/S0090-4295(01)01207-9 11489682

[45] Joosten SC Odeh SNO Koch A Buekers N Aarts MJB Baldewijns M. Development of a Prognostic Risk Model for Clear Cell Renal Cell Carcinoma by Systematic Evaluation of DNA Methylation Markers. Clin Epigenet (2021) 13:103. doi: 10.1186/s13148-021-01084-8 PMC8094610 33947447

[46] Yao ZY Xing CQ Zhang T Liu YW Xing XL. MicroRNA Related Prognosis Biomarkers From High Throughput Sequencing Data of Kidney Renal Papillary Cell Carcinoma. Eur Rev Med Pharmacol Sci (2021) 25:2235–44. doi: 10.26355/eurrev_202103_25255 33755961

[47] Liu Y Gou X Wei Z Yu H Zhou X Li X. Bioinformatics Profiling Integrating a Four Immune-Related Long Non-Coding RNAs Signature as a Prognostic Model for Papillary Renal Cell Carcinoma. Aging (Albany NY) (2020) 12:15359–73. doi: 10.18632/aging.103580 PMC7467365 32716909

[48] Jiang Y Gou X Wei Z Tan J Yu H Zhou X. Bioinformatics Profiling Integrating a Three Immune-Related Long Non-Coding RNA Signature as a Prognostic Model for Clear Cell Renal Cell Carcinoma. Cancer Cell Int (2020) 20:166. doi: 10.1186/s12935-020-01242-7 32435157 PMC7222502

[49] Ye JJ Cheng YL Deng JJ Tao WP Wu L. LncRNA LINC00460 Promotes Tumor Growth of Human Lung Adenocarcinoma by Targeting miR-302c-5p/FOXA1 Axis. Gene (2019) 685:76–84. doi: 10.1016/j.gene.2018.10.058 30359741

[50] Kong YG Cui M Chen SM Xu Y Xu Y Tao ZZ. LncRNA-LINC00460 Facilitates Nasopharyngeal Carcinoma Tumorigenesis Through Sponging miR-149-5p to Up-Regulate IL6. Gene (2018) 639:77–84. doi: 10.1016/j.gene.2017.10.006 28987345

[51] Liang Y Wu Y Chen X Zhang S Wang K Guan X. A Novel Long Noncoding RNA Linc00460 Up-Regulated by CBP/P300 Promotes Carcinogenesis in Esophageal Squamous Cell Carcinoma. Biosci Rep (2017) 37(5):BSR20171019. doi: 10.1042/BSR20171019 28939763 PMC5964888

[52] Lian Y Yan C Xu H Yang J Yu Y Zhou J. A Novel lncRNA, LINC00460, Affects Cell Proliferation and Apoptosis by Regulating KLF2 and CUL4A Expression in Colorectal Cancer. Mol Ther Nucleic Acids (2018) 12:684–97. doi: 10.1016/j.omtn.2018.06.012 PMC6083012 30092404

[53] Zhang Y Liu X Li Q Zhang Y. lncRNA LINC00460 Promoted Colorectal Cancer Cells Metastasis via miR-939-5p Sponging. Cancer Manag Res (2019) 11:1779–89. doi: 10.2147/CMAR.S192452 PMC6391123 30863183

[54] Wang X Mo FM Bo H Xiao L Chen GY Zeng PW. Upregulated Expression of Long Non-Coding RNA, LINC00460, Suppresses Proliferation of Colorectal Cancer. J Cancer (2018) 9:2834–43. doi: 10.7150/jca.26046 PMC6096368 30123352

[55] Ma G Zhu J Liu F Yang Y. Long Noncoding RNA LINC00460 Promotes the Gefitinib Resistance of Nonsmall Cell Lung Cancer Through Epidermal Growth Factor Receptor by Sponging miR-769-5p. DNA Cell Biol (2019) 38:176–83. doi: 10.1089/dna.2018.4462 PMC6383575 30601026

[56] Li K Sun D Gou Q Ke X Gong Y Zuo Y. Long non-Coding RNA Linc00460 Promotes Epithelial-Mesenchymal Transition and Cell Migration in Lung Cancer Cells. Cancer Lett (2018) 420:80–90. doi: 10.1016/j.canlet.2018.01.060 29409808

[57] Yue QY Zhang Y. Effects of Linc00460 on Cell Migration and Invasion Through Regulating Epithelial-Mesenchymal Transition (EMT) in non-Small Cell Lung Cancer. Eur Rev Med Pharmacol Sci (2018) 22:1003–10. doi: 10.26355/eurrev_201802_14382 29509248

[58] Dai C Zhang Y Ni H Kuang Y Xu Z. Prognostic Significance of LINC00460 Overexpression in Solid Tumours: A Meta-Analysis. Postgrad Med J (2020) 96:286–95. doi: 10.1136/postgradmedj-2019-137172 32054779

[59] Luo Y Tan W Jia W Liu Z Ye P Fu Z. The Long non-Coding RNA LINC01606 Contributes to the Metastasis and Invasion of Human Gastric Cancer and is Associated With Wnt/beta-Catenin Signaling. Int J Biochem Cell Biol (2018) 103:125–34. doi: 10.1016/j.biocel.2018.08.012 30142387

[60] Zimta AA Tomuleasa C Sahnoune I Calin GA Berindan-Neagoe I. Long Non-Coding RNAs in Myeloid Malignancies. Front Oncol (2019) 9:1048. doi: 10.3389/fonc.2019.01048 31681586 PMC6813191

