# Supplementary material for: Identification of a Somatic Mutation-Derived Long Non-Coding RNA Signatures of Genomic Instability in Renal Cell Carcinoma
Source: Front Oncol. 2021 Oct 5;11:728181. doi: 10.3389/fonc.2021.728181 (PMC8523920; doi:10.3389/fonc.2021.728181)
Supplement: Supplementary file 1 [file DataSheet_1.docx]

Supplementary Material

## Supplementary Figures


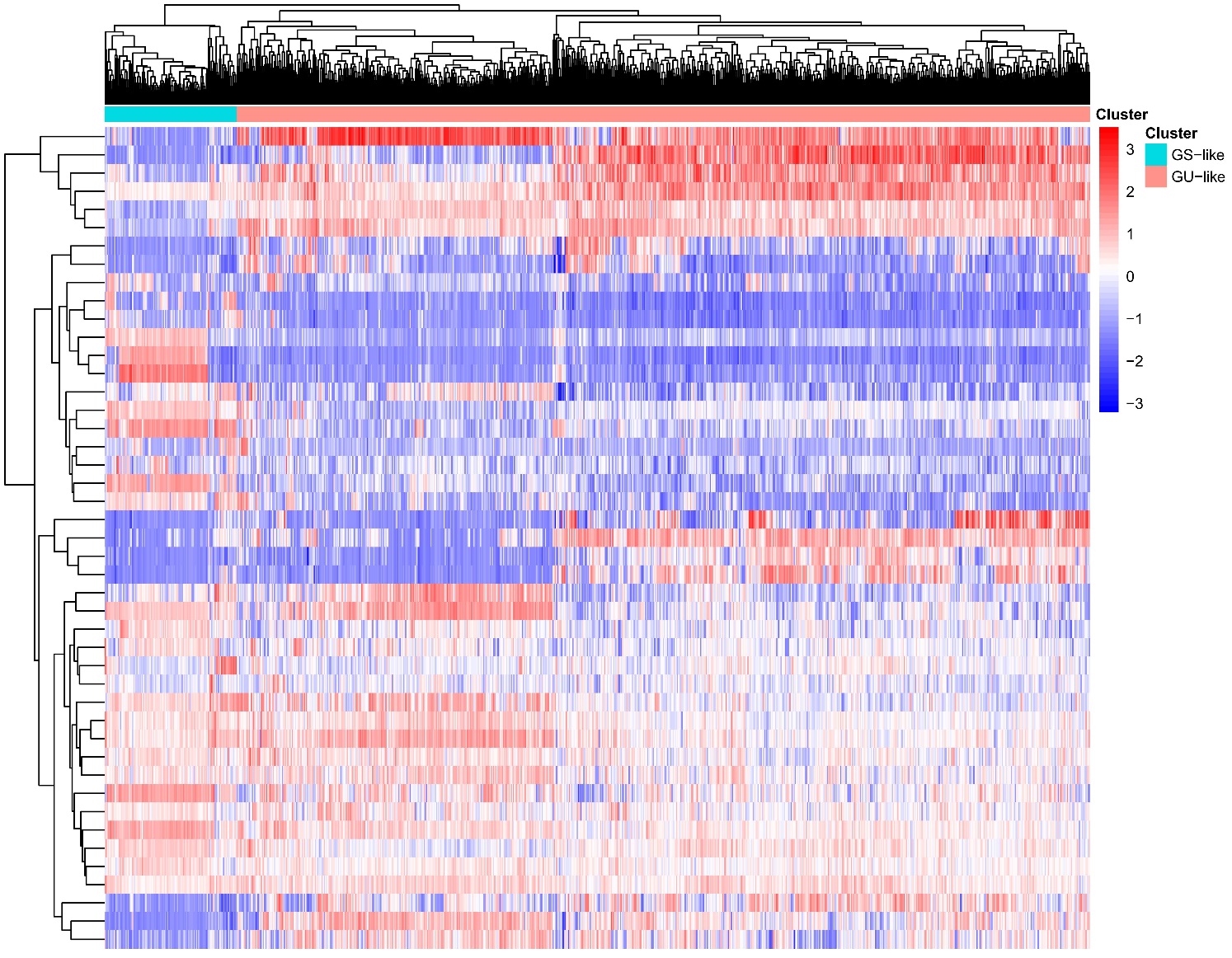


**Supplementary Figure 1.** **Differentially expressed lncRNAs in Gene Unstable (GU) group and Gene Stable (GS) group of renal cell carcinoma(RCC) patients.** Data was retrieved from TCGA database. The GU group and the GS group each contained 85 samples, and a total of 45 lncRNAs with statistical differences (p< 0.05).

**1.2 Supplementary Tables**

**Supplementary Table 1 LncRNAs significantly associated with overall survival from univariate Cox analysis in the Train set.**

| **id** | **HR** | **HR.95L** | **HR.95H** | **P value** |
| --- | --- | --- | --- | --- |
| MCF2L-AS1 | 0.90076767 | 0.814104 | 0.99665694 | 0.04288334 |
| LINC02471 | 0.98455861 | 0.97106519 | 0.99823953 | 0.02708981 |
| LINC00460 | 1.12973028 | 1.08252582 | 1.17899313 | 0.00000002 |
| AC148477.4 | 0.96816717 | 0.94062640 | 0.99651431 | 0.02801250 |
| KRT7-AS | 0.83888205 | 0.71370898 | 0.98600846 | 0.03310034 |
| AC156455.1 | 1.13394402 | 1.08165880 | 1.18875660 | 0.00000018 |
| AC015977.2 | 1.20943795 | 1.04405427 | 1.40101926 | 0.01125758 |
| AC243964.3 | 0.76258007 | 0.60292350 | 0.96451435 | 0.02373394 |
| LINC01234 | 1.12934280 | 1.03577513 | 1.23136299 | 0.00584158 |
| AL031123.1 | 0.73900132 | 0.58420286 | 0.93481732 | 0.01166864 |
| PRDM16-DT | 0.89555432 | 0.81023604 | 0.98985665 | 0.03080744 |
| AL139351.1 | 1.17760100 | 1.04039742 | 1.33289845 | 0.00969405 |
| AC114803.1 | 1.04116450 | 1.00251460 | 1.08130447 | 0.03661001 |
| AC103563.7 | 0.83059125 | 0.74945281 | 0.92051403 | 0.00040144 |
| AL035661.1 | 0.97316500 | 0.95200187 | 0.99479858 | 0.01531489 |
| AC144831.1 | 0.81942105 | 0.68300938 | 0.98307707 | 0.03205878 |
| LINC01187 | 0.98832991 | 0.97856046 | 0.99819689 | 0.02055622 |
| AC005082.1 | 0.83223812 | 0.73204042 | 0.94615034 | 0.00502098 |
| AC079466.1 | 1.00991970 | 1.00208099 | 1.01781973 | 0.01303311 |
| ZNF582-AS1 | 0.73715128 | 0.56203476 | 0.96682989 | 0.02754376 |
| LINC01606 | 1.03320163 | 1.00091224 | 1.06653267 | 0.04377375 |
| AL031710.1 | 0.94789944 | 0.91280051 | 0.98434799 | 0.00544514 |
| GAS6-DT | 0.84894569 | 0.73324612 | 0.98290160 | 0.02847546 |
| ST3GAL6-AS1 | 0.77396581 | 0.60516429 | 0.98985199 | 0.04122892 |
| AC130371.2 | 0.81455676 | 0.70086709 | 0.94668835 | 0.00748942 |

**Supplementary Table 2 LncRNAs significantly associated with overall survival from multivariate Cox analysis in the Train set.**

| **id** | **coef** | **HR** | **HR.95L** | **HR.95H** | **P value** |
| --- | --- | --- | --- | --- | --- |
| LINC00460 | 0.085 | 1.088 | 1.036 | 1.143 | 0.001 |
| AC156455.1 | 0.213 | 1.238 | 1.111 | 1.379 | 0.000 |
| AC015977.2 | 0.195 | 1.216 | 1.032 | 1.432 | 0.019 |
| AL031123.1 | -0.253 | 0.777 | 0.562 | 1.072 | 0.125 |
| `PRDM16-DT` | 0.218 | 1.244 | 0.996 | 1.554 | 0.055 |
| AL139351.1 | 0.172 | 1.188 | 1.027 | 1.374 | 0.020 |
| AC114803.1 | -0.096 | 0.909 | 0.847 | 0.974 | 0.007 |
| AC103563.7 | -0.155 | 0.856 | 0.756 | 0.970 | 0.015 |
| AL035661.1 | 0.054 | 1.056 | 1.002 | 1.112 | 0.043 |
| LINC01606 | 0.087 | 1.091 | 1.039 | 1.146 | 0.000 |
| AL031710.1 | -0.139 | 0.871 | 0.754 | 1.005 | 0.059 |
